# Viral regulation of host cell biology by hijacking of the nucleolar DNA-damage response

**DOI:** 10.1038/s41467-018-05354-7

**Published:** 2018-08-03

**Authors:** Stephen M. Rawlinson, Tianyue Zhao, Ashley M. Rozario, Christina L. Rootes, Paul J. McMillan, Anthony W. Purcell, Amanda Woon, Glenn A. Marsh, Kim G. Lieu, Lin-Fa Wang, Hans J. Netter, Toby D. M. Bell, Cameron R. Stewart, Gregory W. Moseley

**Affiliations:** 1 Department of Microbiology, Biomedicine Discovery Institute, Monash University, Clayton, Victoria, 3800 Australia; 2 Department of Biochemistry and Molecular Biology, Bio21 Institute, The University of Melbourne, Melbourne, Victoria, 3010 Australia; 3 School of Chemistry, Monash University, Clayton, Victoria, 3800 Australia; 4 CSIRO Health & Biosecurity, Australian Animal Health Laboratory, Geelong, Victoria, 3220 Australia; 5 Biological Optical Microscopy Platform, The University of Melbourne, Parkville, Victoria, 3010 Australia; 6 Infection and Immunity Program, Biomedicine Discovery Institute, Monash University, Clayton, Victoria, 3800 Australia; 7 Department of Biochemistry and Molecular Biology, Monash University, Clayton, Victoria, 3800 Australia; 8 Programme in Emerging Infectious Diseases, Duke-NUS Medical School, Singapore, 169857 Singapore; 9 Victorian Infectious Diseases Reference Laboratory, Melbourne Health, The Peter Doherty Institute, Victoria, 3000 Australia

## Abstract

Recent studies indicate that nucleoli play critical roles in the DNA-damage response (DDR) via interaction of DDR machinery including NBS1 with nucleolar Treacle protein, a key mediator of ribosomal RNA (rRNA) transcription and processing. Here, using proteomics, confocal and single molecule super-resolution imaging, and infection under biosafety level-4 containment, we show that this nucleolar DDR pathway is targeted by infectious pathogens. We find that the matrix proteins of Hendra virus and Nipah virus, highly pathogenic viruses of the Henipavirus genus in the order *Mononegavirales*, interact with Treacle and inhibit its function, thereby silencing rRNA biogenesis, consistent with mimicking NBS1–Treacle interaction during a DDR. Furthermore, inhibition of Treacle expression/function enhances henipavirus production. These data identify a mechanism for viral modulation of host cells by appropriating the nucleolar DDR and represent, to our knowledge, the first direct intranucleolar function for proteins of any mononegavirus.

## Abstract (web summary)

Many RNA viruses that replicate in the cytoplasm express proteins that localize to nucleoli, but the nucleolar functions remain largely unknown. Here, the authors show that the Henipavirus matrix protein mimics an endogenous Treacle partner of the DNA-damage response, resulting in suppression of rRNA biogenesis.

## Introduction

The DNA-damage response (DDR) comprises a complex network of pathways that monitor and repair damage to genomic DNA to prevent deleterious mutations^1^. The mechanisms underlying the DDR are only partially resolved, but recent studies implicated nucleoli as having critical roles^2–5^. The canonical function of nucleoli is ribosome biogenesis, where they support ribosomal RNA (rRNA) synthesis, processing, and assembly into pre-ribosomal subunits. However, numerous studies have indicated that nucleoli are highly multifunctional and dynamic structures involved in processes including stress responses, cell-cycle regulation and signal recognition particle assembly^6^. These functions derive from a large nucleolar proteome, by which nucleoli are considered to act as integrators of complex cellular signals, the full extent and mechanisms of which are only beginning to be understood^6^.

Recent studies of the roles of nucleoli in stress responses have identified Treacle protein, a nucleolar regulator of rDNA transcription and pre-rRNA processing that localizes to subnucleolar compartments, as a critical mediator of rRNA silencing that is induced by the DDR^2,4,7–10^. Specifically, DDR-induced rRNA silencing involves Nijmegen Breakage Syndrome 1 (NBS1) protein, a key effector protein of the DDR that forms part of the MRN complex^1^. NBS1 interacts with Treacle and, during a DDR, accumulates in Treacle-enriched compartments to induce rRNA synthesis silencing^2,4^. Under normal conditions, Treacle maintains basal levels of rRNA biogenesis such that depletion of Treacle expression results in reduced rRNA synthesis^4,9^. During the DDR, the extent of rRNA inhibition observed is equivalent to that following Treacle depletion, and induction of the DDR in cells depleted for Treacle causes no additional inhibition^4^. Thus, DDR-induced silencing of rRNA synthesis is Treacle-dependent, and the data appear consistent with a model whereby Treacle’s normal function in rRNA biogenesis is inhibited via the NBS1–Treacle complex; however, the precise mechanism has not been confirmed^2,4^. Notably, reduced Treacle expression and consequent effects on rRNA biogenesis are associated with the genetic disorder Treacher-Collins Syndrome (TCS), a severe craniofacial developmental disorder wherein mutations of the Treacle-encoding *TCOF1* gene account for the majority of cases^11^. Haplo-insufficiency of Treacle is thought to result in insufficient ribosome biogenesis in highly proliferative neuroepithelial cells during development, leading to nucleolar stress and activation of apoptosis^12^.

Other than its roles in genetic disorders such as TCS, neurodegenerative diseases^13^, and cancers^14^, the nucleolus is targeted by proteins expressed by diverse viruses, potentially enabling viral modulation of intranucleolar processes controlling host cell biology^15,16^. However, this aspect of viral biology remains poorly characterized, particularly with respect to viruses of the order *Mononegavirales*, which comprises non-segmented negative-strand RNA viruses including the highly pathogenic Hendra (HeV) and Nipah (NiV) viruses (genus *Henipavirus*, family *Paramyxoviridae*), rabies virus, Ebola virus, measles virus, and mumps virus. Almost all mononegaviruses mediate transcription, replication, and assembly exclusively within the cytoplasm, but several recent reports indicate that certain proteins of mononegaviruses can localize to the nucleolus and bind to specific nucleolar proteins^17–19^.

The henipavirus matrix (M) protein is perhaps the best characterized of the nucleolar proteins expressed by mononegaviruses. The key henipavirus members are HeV and NiV, which are zoonotic viruses that have emerged from bat reservoirs to cause multiple outbreaks in humans and domesticated animals, with mortality rates between 40 and 75%, and no human vaccine or therapeutic commercially available^20^. Henipavirus M protein plays essential roles in virus particle assembly in the cytoplasm and budding at the plasma membrane, and can self-assemble into secretion-competent virus-like particles (VLPs)^21,22^; these functions are broadly conserved among paramyxoviruses and other mononegaviruses^23,24^. However, M protein also enters the nucleus and accumulates within nucleoli in NiV- and HeV-infected cells^19,25^. Recent studies reported a requirement for M protein to traffic to the nucleolus prior to fulfilling its role in budding^19,26^. Furthermore, certain proteomic data suggest that M protein can interact with multiple nucleolar factors, supporting the possibility of an intranucleolar role^26,27^. However, it has also been proposed that the principal role of nuclear/nucleolar localization of proteins of mononegaviruses might be to sequester inhibitory proteins from the cytoplasm during replication prior to budding^15,24,28^, and importantly, no direct nucleolar function has been reported for M protein or for nucleolar proteins expressed by any mononegavirus; thus, the potential significance of such interactions remains unresolved.

Here, we show that Henipavirus M proteins specifically targets Treacle protein and inhibits rRNA biogenesis. The data indicate that M protein enters Treacle-enriched compartments in the nucleolus and exploits the DDR-Treacle pathway by a mechanism consistent with mimicking DDR-activated NBS1. This identifies a novel viral strategy to subvert the biology of the host cell.

## Results

### HeV M protein accumulates in a subnucleolar compartment

**Fig. 1:**
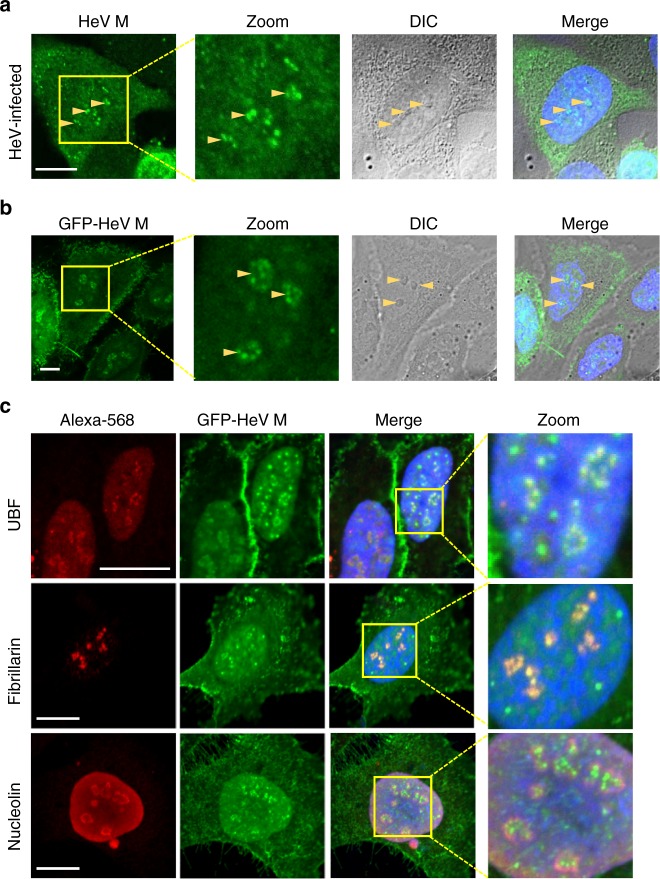
HeV M protein localizes to discrete subnucleolar compartments in HeV-infected cells and cells transfected to express HeV M protein. **a** HeLa cells infected with HeV (multiplicity of infection (MOI) of 5) were fixed 24 h post-infection (p.i.) and immunostained for HeV M protein before imaging by CLSM. **b** HeLa cells transfected to express GFP-HeV M protein were imaged live 16 h post-transfection (p.t.) by CLSM. Images are representative of ≥5 independent experiments, in each of which ≥10 fields of view were captured. **c** HeLa cells transfected to express GFP-HeV M protein were fixed 16 h p.t. and immunolabeled for the indicated nucleolar proteins, using AlexaFluor-568-conjugated secondary antibodies. Images are representative of cells in ≥20 fields of view. Arrowheads indicate nucleoli. Nuclei (DNA) were labeled using Hoechst 33342 (blue in merged images). Yellow boxes highlight regions of images magnified in the Zoom panel. Scale bars correspond to 15 μm

Nucleoli comprise multiple compartments with discrete functions such that protein localization to particular subnucleolar regions enables different regulatory roles^29,30^. The nucleolus is classically divided into three major subcompartments, the inner fibrillar center (FC), dense fibrillar component (DFC) and granular component (GC), which appear to mediate specific stages of ribosome biogenesis through distinct proteomes. Although HeV M protein is known to target the nucleolus^17,19,26^ its specific subnucleolar localization has not been described. To examine this, we used confocal laser scanning microscopy (CLSM) to image nucleoli in cells infected by HeV and immunostained for M protein (Fig. 1a), or in living cells transfected to express HeV M protein fused to green fluorescent protein (GFP-HeV M) (Fig. 1b and Supplementary Movie 1). Rather than being diffuse within nucleoli, M protein accumulated strongly within discrete subnucleolar compartments in both infected and transfected cells, indicating an intrinsic capacity of M protein to interact with specific compartments, which is independent of other viral proteins. Immunofluorescence (IF) labeling for nucleolar markers indicated co-localization of HeV M with compartments containing UBF (a marker for FC/DFC) and FBL (a marker for DFC), while NCL (a marker for GC) appeared to surround HeV M compartments (Fig. 1c)^29–31^. Thus, the subnucleolar compartments containing HeV M likely correspond to FC/DFC.

### K258A mutation of M prevents subnucleolar accumulation

Henipavirus M protein contains at least one  nuclear localization sequence (NLS) and one  nuclear export sequence (NES)^19,21^, but the requirements for nucleolar targeting are unknown. Since NLSs and nucleolar localization sequences (NoLSs) are often proximal or overlap^32^ we examined the impact on nucleolar localization of mutation of K258 to alanine (K258A), which was reported to inhibit nuclear localization of NiV M protein, suggesting that K258 forms part of a C-terminal NLS^19,26^.

**Fig. 2:**
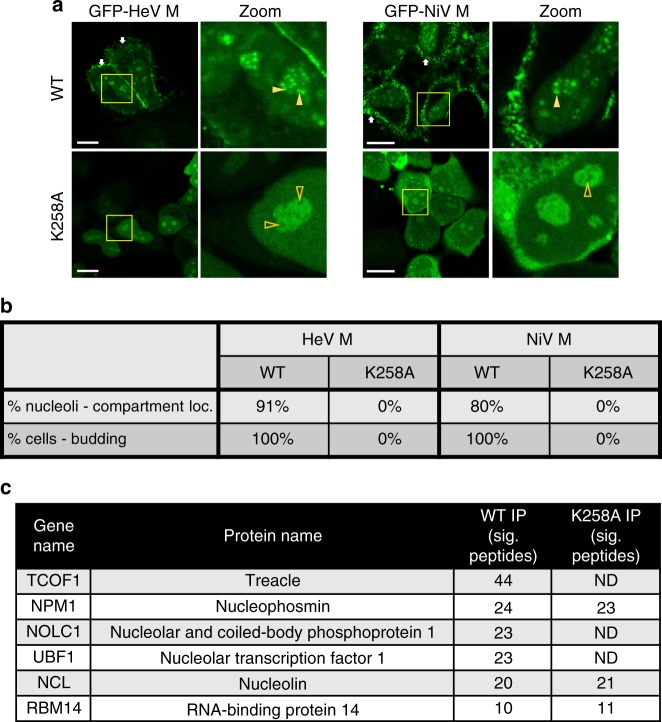
Mutation of residue K258 to alanine inhibits localization to subnucleolar compartments, binding to a subset of nucleolar proteins, and budding function of M protein. **a** HeLa cells transfected to express the indicated proteins were analyzed by live-cell CLSM; white arrows indicate localization of M protein at the plasma membrane/membrane protrusions; filled and unfilled yellow arrowheads indicate subnucleolar compartments with apparent accumulation and exclusion, respectively, of M protein. Scale bars correspond to 15 μm. **b** Images such as those in **a** were used to determine the percentage of nucleoli with clear accumulation of M protein in subnucleolar compartments (% nucleoli—compartment loc.; ≥447 nucleoli analyzed for each of WT and K258A-mutated HeV M protein, from four separate assays; ≥99 nucleoli analyzed for each of WT and K258A-mutated NiV M protein, from two separate assays), and the percentage of cells with localization of M protein to concentrated patches at the plasma membrane and into membrane protrusions, consistent with budding (% cells—budding; ≥83 cells analyzed for each sample from at least two independent experiments). **c** A subset of nucleolar interactors of GFP-HeV M and GFP-HeV M-K258A identified by IP/MS; proteins are ranked by number of significant (sig.) peptides identified; ND not detected

GFP-fused M proteins of HeV and NiV showed a similar phenotype, with clear nuclear and nucleolar localization, and accumulation into subnucleolar compartments in c. 91% (HeV M protein) and 80% (NiV M protein) of nucleoli examined (Fig. 2a, b). Both proteins also displayed localization to the plasma membrane/membrane extensions in 100% of cells, consistent with previous observations for NiV M and known roles in budding^19^, which results in VLP formation in M-protein-expressing cells^19,21^. Mutation of K258 to A did not prevent accumulation of HeV or NiV M protein within nucleoli, but strongly impaired accumulation within subnucleolar compartments, such that no compartmental localization was observed in nucleoli of cells expressing mutated HeV or NiV M proteins (Fig. 2a, b). This suggested that mutation of K258 inhibits localization of M protein to subnucleolar compartments and, consistent with this, we were able to detect clear exclusion of K258A-mutated M protein from regions consistent with these compartments (see Zoom panels Fig. 2). As previously reported for NiV M^19^, the membrane localization/extension phenotype was entirely prevented by K258A mutation, and this effect was also observed for HeV M protein (Fig. 2a, b) indicating impaired budding function. This was directly confirmed using VLP budding assays (Supplementary Fig. 1A).

Interestingly, despite similar effects of K258A mutation on the localization of HeV M and NiV M proteins to subnucleolar compartments and plasma membrane, K258A had markedly different effects on the nuclear localization of these proteins, producing a more cytoplasmic localization of NiV M (as previously reported^19^), but having little to no effect on nucleo-cytoplasmic localization of HeV M protein (Fig. 2)^19^. Thus, it appears that the requirements for subnucleolar localization and budding are conserved between HeV and NiV M proteins, while those for nucleo-cytoplasmic localization differ, in spite of high sequence conservation between the proteins (c. 90% amino acid identity). Importantly, the finding that K258A mutation did not prevent nucleolar localization of HeV M protein, but resulted in a failure to accumulate within subnucleolar compartments, provided the opportunity to examine the specific roles and interactions associated with subnucleolar localization.

### M protein interacts with Treacle protein in compartments

To identify cellular factors associated with the subnucleolar localization of M protein we performed comparative immunoprecipitation/mass spectrometry (IP/MS) to compare the prevalence of nucleolar proteins in the interactome of wt and K258A-mutated GFP-HeV M proteins. The most pronounced nucleolar interactors of wt M protein, according to the number of significant peptides, included nucleolar proteins Treacle, nucleolar and coiled-body phosphoprotein 1 (NOLC1) and UBF1 (Fig. 2c and Supplementary Data 1). Notably, none of these proteins were detected in IP/MS in the interactome of K258A-mutated HeV M protein. Since wt and K258A-mutated HeV M protein interacted similarly with other nucleolar proteins, including nucleophosmin (NPM1), NCL and RNA-binding protein 14 (RMB14), it appeared that K258A specifically impacts interactions with a subset of nucleolar proteins, indicating that these interactors are relevant to subnucleolar compartment localization.

**Fig. 3:**
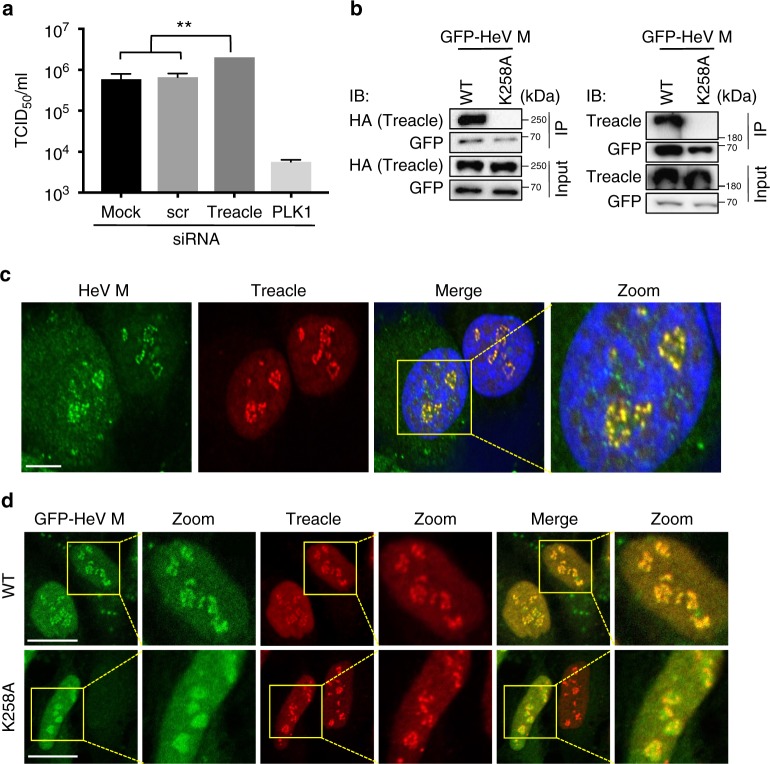
Treacle impacts HeV production and interacts with wt but not K258A HeV M protein. **a** HeLa cells were transfected with scrambled siRNA (scr), Treacle siRNA, or mock-transfected, before infection with HeV (MOI 0.5); siRNA for polo-like kinase 1 (PLK1) is a positive control known to inhibit HeV production^17^. HeV titer was measured at 48 h p.i. (mean TCID_50_/mL, ± s.e.m., *n* = 3). Statistical analysis used Student’s *t*-test; **, *p* = 0.003. **b** IPs of the indicated proteins from HEK-293T cells were analyzed by immunoblotting (IB) with the indicated antibodies; results are representative of three independent experiments. **c** HeV-infected cells were fixed 24 h p.i. and immunostained for HeV M protein and Treacle. Nuclei were labeled using Hoechst 33342 (blue in merged image). Images are representative of ≥20 fields of view, capturing >100 cells over two experiments. **d** HeLa cells transfected to express the indicated proteins were fixed and immunostained for Treacle; images are representative of ≥30 fields of view over three experiments. Scale bars correspond to 15 μm

The most pronounced nucleolar interactor was Treacle, which is known to localize to a subnucleolar compartment, where, in common with HeV M (Figs. 1, 2), it colocalizes with factors such as FBL indicative of association with FC/DFC^33^. We thus selected Treacle for further analysis. To assess the impact of Treacle on the production of infectious HeV, we transfected cells with control (scr) siRNA or siRNA targeting Treacle, before infection with HeV. The depletion of Treacle expression (Supplementary Fig. 2) resulted in a significant increase in virus production (Fig. 3a). These data suggested that M protein might target Treacle to suppress its function to benefit the virus, such that depletion of Treacle enhances this proviral effect. Alternatively, Treacle might have intrinsic antiviral function through binding to M protein, which is relieved by Treacle depletion.

**Fig. 4:**
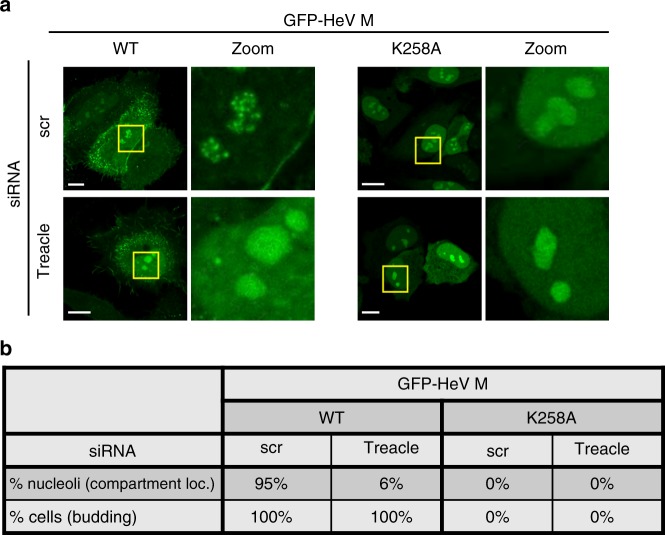
Subnucleolar compartment localization, but not budding function of M protein, is dependent on Treacle. **a** HeLa cells were transfected with scr siRNA or Treacle siRNA two days prior to transfection to express the indicated GFP-HeV M proteins, and imaging of living cells 16 h later. Representative images are shown for each condition; yellow boxes highlight regions of images magnified in the Zoom panel. Scale bars correspond to 15 μm. **b** Images such as those in **a** were used to calculate the percentage of nucleoli with subnucleolar compartment localization (% nucleoli—compartment loc.; analysis of ≥99 nucleoli for each condition from three independent experiments) and percentage of cells with ‘budding’ phenotype, as described in legend to Fig. 2 (% cells—budding; ≥91 cells analyzed per sample from three independent experiments)

Using IP and immunoblot (IB) analysis, we further confirmed that GFP-HeV M interacts with endogenous Treacle and with co-transfected HA-Treacle, and that mutation of K258 prevents these interactions (Fig. 3b). Similar analysis indicated a lack of any effect of K258A mutation on interaction of HeV M with FBL (Supplementary Fig. 3), which was previously shown to bind HeV M^17^; this confirmed the specificity of the effect of K258A on interaction with certain nucleolar proteins. Co-localization of HeV M with endogenous Treacle within the subnucleolar compartments was also confirmed by CLSM analysis of infected cells (Fig. 3c) and cells expressing GFP-HeV M (Fig. 3d upper panels). HeV M-K258A did not accumulate with Treacle in compartments (Fig. 3d lower panels). Notably, siRNA depletion of Treacle also inhibited the compartmental accumulation of GFP-HeV M (Fig. 4a, b). Thus, it appears that M protein interacts with Treacle protein in Treacle-enriched subnucleolar compartments^33^.

**Fig. 5:**
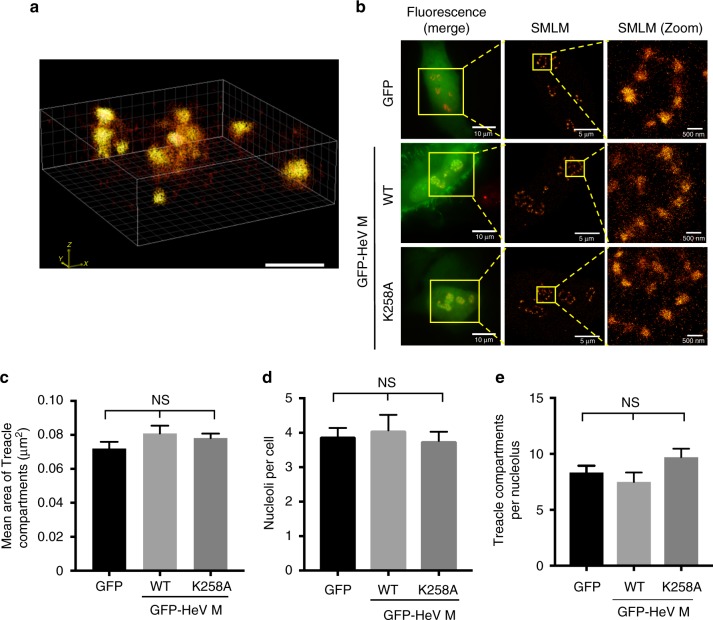
HeV M protein expression does not impact the structure or distribution of Treacle-enriched compartments. HeLa cells expressing GFP alone (**a**) or the indicated proteins (**b**) were fixed and immunolabeled for Treacle before analysis. **a** *d*STORM single molecule localization microscopy (SMLM) was used to generate 3D images of Treacle-enriched subnucleolar compartments. Scale bar indicates 1 μm. **b** Fluorescence microscopy was used to identify cells expressing GFP (green) and immunostained for Treacle (red/yellow in merged image), which were then analyzed by SMLM to detect Treacle. Scale bars correspond to the indicated sizes. **c**–**e** Images such as those shown in Fig. 5b were used to determine (**c**) the area of Treacle-enriched compartments (mean ± s.e.m., *n* ≥ 427 compartments), (**d**) the number of nucleoli per cell (mean ± s.e.m., *n* ≥ 14 cells), (**e**) the number of Treacle-enriched compartments per nucleolus (mean ± s.e.m., n ≥ 57 nucleoli). Statistical analysis used Student’s *t*-test; NS not significant

To determine whether HeV M interaction with the Treacle-enriched compartments results in gross effects on their structure or distribution, we employed super-resolution microscopy using single molecule localizations (*d*STORM)^34,35^ to analyze cells immunostained for Treacle. This achieved spatial resolution at least as good as 40 nm in *xy* and 80 nm in *z*, enabling, to our knowledge, the first super-resolution measurement of the dimensions of Treacle-containing compartments within the nucleolus. 3D super-resolution images indicated that the compartments are largely spheroidal structures (Fig. 5a and Supplementary Movie 2), with axial cross-sections similar to the feature size detected in 2D super-resolution images (Fig. 5b), for which the mean area of compartments was 0.077 µm^2^ (*n* = 1959 subnucleolar compartments in 226 nucleoli, 59 cells). Importantly, the mean area was not significantly affected by expression of wt or mutated GFP-HeV M protein (Fig. 5c), compared with expression of GFP alone. Furthermore, there was no difference in the mean number of nucleoli per cell (Fig. 5d), or compartments per nucleolus (Fig. 5e) detected by *d*STORM. Thus, HeV M protein does not appear to affect either the number of Treacle-enriched subnucleolar compartments or their dimensions, such that any functional effect of HeV M protein subnucleolar localization is likely to result from specific intranucleolar protein interactions.

### Treacle interaction and budding function of M are distinct

It has been proposed that M protein enters nuclei/nucleoli prior to transport to the plasma membrane for budding, dependent on K258^24^. Since K258A mutation of HeV M protein impacted both Treacle binding/localization to subnucleolar compartments and budding at the plasma membrane, we tested whether these processes are linked by examining effects of Treacle depletion on budding. In contrast to K258A mutation, which entirely prevented budding, Treacle knockdown had no apparent inhibitory effect on budding in microscopy or VLP assays (Fig. 4b and Supplementary Fig. 1B). As described above, Treacle depletion and K258A mutation had comparable effects on subnucleolar localization. Thus, it appears that M protein-Treacle interaction and M protein-mediated budding are distinct processes, suggesting that K258 is important in at least two independent functions in HeV M, as well as impacting nuclear localization in NiV M.

### M inhibits rRNA biogenesis by hijacking the nucleolar DDR

**Fig. 6:**
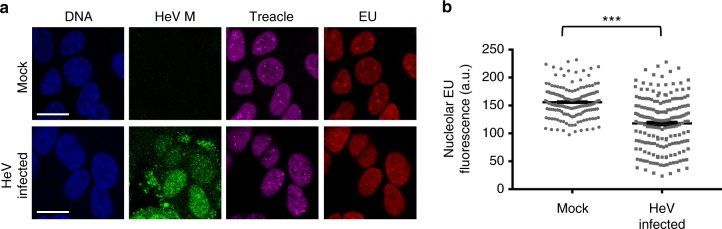
rRNA production is inhibited in HeV infected cells. **a** HeLa cells were mock- or HeV-infected for 23 h before incubation with EU (1 h), fixation and processing to label nascent RNA, as described in Materials and Methods. Cells were then labeled using antibodies to HeV M and Treacle proteins, and Hoechst 33342 (DNA) and then imaged by CLSM. Scale bars correspond to 15 μm. **b** Images such as those in **a** were analyzed to quantify rRNA production (histogram shows mean nucleolar EU fluorescence; ≥156 nucleoli analyzed for each condition over two biological replicates). Statistical analysis used Student’s *t*-test; ***, *p* < 0.001

**Fig. 7:**
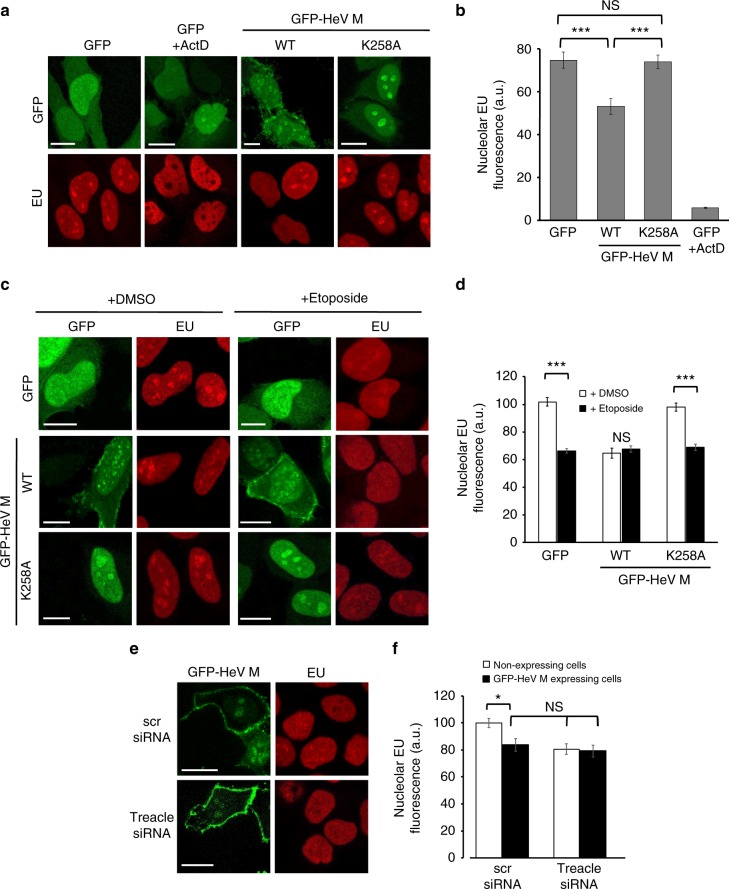
HeV M protein expression inhibits rRNA production. HeLa cells were transfected to express the indicated GFP-fused proteins before treatment with or without ActD for 1 h (**a**, **b**), or DMSO or etoposide (3 h) (**c**, **d**), and labeling of nascent RNA as described in the legend to Fig. 6 (fixation at 16 h p.t.; histogram shows mean nucleolar EU fluorescence ± s.e.m.; *n* ≥ 31 nucleoli, data from a single assay, representative of six independent assays for **b**; *n* ≥ 39 nucleoli, data from a single assay, representative of three independent assays for **d**). **e**, **f** HeLa cells were transfected with scrambled (scr) or Treacle siRNA followed by transfection to express GFP-HeV M protein and labeling of nascent RNA (fixation 16 h p.t.; histogram shows mean nucleolar EU fluorescence ± s.e.m., *n* ≥ 23 nucleoli, data from a single assay representative of three independent assays). Statistical analysis used Student’s *t*-test; * *p* < 0.05; **, *p* < 0.01; *** *p* < 0.001; NS, Non-significant. Scale bars correspond to 15 μm

The above data indicate that M protein-Treacle interaction does not affect the classical role of M protein in virus budding, suggesting that M protein targets Treacle to modulate host cell function. Treacle regulates rRNA production and is required for efficient ‘basal’ synthesis of rRNA, providing a molecular target for cellular regulation of rRNA, as suggested by its role in the DDR^2,4,9^. To examine whether M protein targets Treacle-enriched compartments to exploit such a regulatory mechanism, we assessed the effect of HeV infection on rRNA synthesis, by performing in situ detection of rRNA in the nucleolus using the Click-iT^TM^ RNA Imaging Kit, which was previously used to identify the role of Treacle in the DDR^4^. A significant (*p* < 0.0001) reduction in rRNA synthesis was observed in HeV-infected cells compared with mock-infected cells (Fig. 6a, b) indicating that HeV infection can inhibit a Treacle-related function. Expression of GFP-HeV M alone also significantly (*p* < 0.001) reduced rRNA synthesis, with the extent of reduction (c. 35%, Fig. 7a, b) similar to that reported following knockdown of Treacle^4^. Notably, treatment of cells with actinomycin D (ActD), which arrests rRNA synthesis globally by inhibiting RNA Polymerase I (RNA Pol I), reduced rRNA production to a greater extent (>90% reduction of rRNA synthesis, Fig. 7b). Thus, the effect of HeV infection and HeV M protein expression is consistent with specific inhibition of Treacle-dependent processes. Consistent with this idea, no effect on rRNA production was observed following expression of GFP-HeV M-K258A, which does not bind to Treacle (Fig. 7a, b).

Since the effect of HeV M protein on rRNA biogenesis was consistent with that observed during a DDR^4^, we sought to define whether the same molecular pathway is being utilized by testing the impact of HeV M protein expression on the inhibition of rRNA synthesis that is induced by DNA damage. Using etoposide, we confirmed that DNA damage inhibits rRNA biogenesis, and found that it does so to a similar extent as HeV M protein expression (Fig. 7c, d). Importantly, combination of these stimuli produced no additional effect, suggesting that HeV M exploits the same pathway of rRNA silencing that occurs during the DDR, which is Treacle-dependent^2,4^ (Fig. 7c, d). To directly examine the role of Treacle in HeV M protein-mediated suppression of rRNA biogenesis, we analyzed rRNA synthesis in cells depleted of Treacle (as above) and/or transfected to express HeV M protein, detecting no additive effect by combining these stimuli (Fig. 7e, f). Together, these data confirm that the effect of M protein on rRNA biogenesis is Treacle-dependent, and, furthermore, indicate that viral subversion of this process is highly potent, being equivalent to the effect observed following either genuine DNA damage or depletion of Treacle.

### DNA damage is induced by HeV infection but not by M protein

**Fig. 8:**
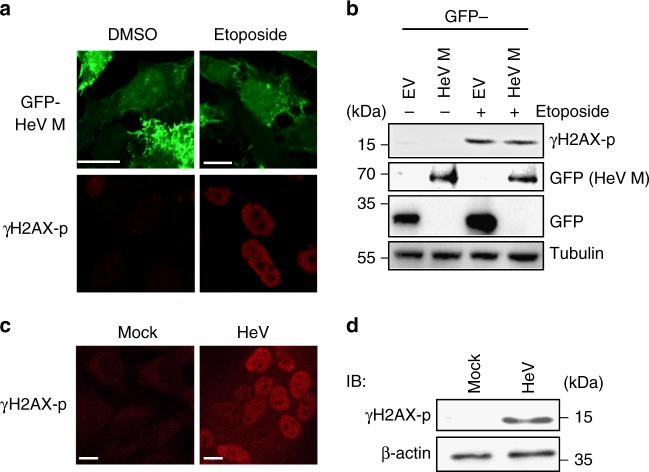
HeV infection but not HeV M protein expression induces DNA damage. **a** HeLa cells transfected to express GFP-HeV M protein without (DMSO) or with etoposide treatment were fixed 16 h p.t., immunostained for γH2AX phosphorylated at S139 (γH2AX-p) and imaged by CLSM. Images are representative of ≥15 fields of view. **b** Lysates of HEK-293T cells transfected to express GFP alone (empty vector (EV)) or GFP-HeV M protein and treated at 16 h p.t. without or with etoposide (3 h), were analyzed by IB using the indicated antibodies (data are representative of two independent experiments). **c** HeLa cells infected with HeV (MOI 5; which results in c. 100% cells infected) were fixed 24 h p.i. and immunostained for γH2AX-p prior to imaging by CLSM (images representative of ≥15 fields of view from three biological replicates). **d** Lysates of mock or HeV-infected HeLa cells were collected at 24 h p.i. and analyzed by IB using the indicated antibodies (data representative of three biological replicates). Scale bars correspond to 15 μm

Our data indicated that HeV M protein specifically activates the nucleolar DDR via interaction with Treacle. To confirm that the effect of M protein is independent of DNA damage (i.e., that M protein expression does not cause DNA damage), we measured the effect of GFP-HeV M protein expression on phosphorylation of the histone variant, γH2AX, at residue S139 (γH2AX-p, a standard indicator of DNA damage). Analysis of IF-labeled cells by CLSM, or of cell lysates by IB (Fig. 8a, b), clearly indicated an increase in γH2AX-p following treatment with etoposide, but no increase in γH2AX-p could be detected following expression of GFP-HeV M protein (Fig. 8a, b), despite comparable effects of wt HeV M protein expression and etoposide treatment on rRNA production (Fig. 7c, d). Thus, it appears that M protein affects the nucleolar DDR via interaction with Treacle rather than through an indirect effect involving DNA damage.

One possible explanation for our finding that HeV M protein modulates the nucleolar DDR is that HeV infection might induce DNA damage, such that alteration of specific DDR pathways/responses by M protein might protect the infected cell from deleterious effects. We thus examined the effect of HeV infection on γH2AX, identifying a clear increase in levels of γH2AX-p levels, suggesting induction of DNA damage (Fig. 8c, d).

### HeV M protein disrupts Treacle–NBS1 complexes

**Fig. 9:**
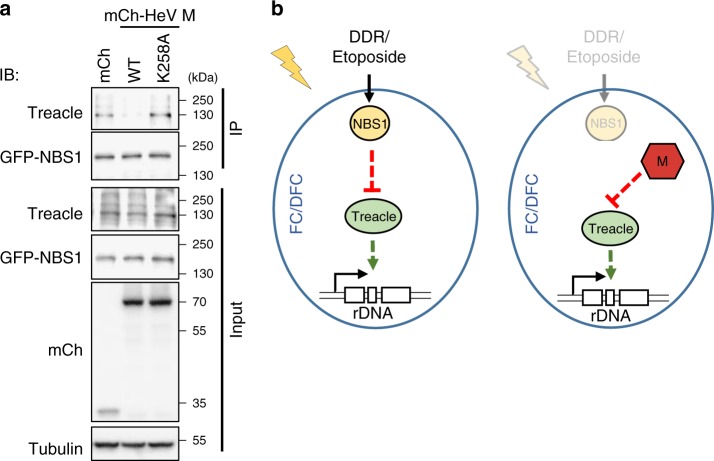
HeV M protein disrupts the Treacle–NBS1 complex. **a** HEK-293T cells co-transfected to express the indicated mCherry (mCh) protein with NBS1-GFP were subjected to IP for NBS1-GFP followed by analysis by IB using the indicated antibodies; results are representative of 3 independent experiments. **b** Models for (left panel) DDR-mediated and (right panel) HeV M protein-mediated inhibition of rRNA synthesis. Treacle is required for basal rRNA synthesis and localizes to Treacle-enriched subnucleolar compartments which appear to correspond to FC/DFC. (Left panel) During the DDR, Treacle function is inhibited by NBS1, with which Treacle forms a complex. (Right panel) During HeV infection, HeV M localizes to FC/DFC and binds to Treacle resulting in inhibition of Treacle-dependent function independently of a DDR; the capacity of M protein to disrupt the complex (**a**) suggests that it binds at or close to the Treacle–NBS1 interaction site to mimic a DDR-activated NBS1–Treacle complex

The properties of HeV M protein with respect to Treacle are analogous to those of DDR-activated NBS1, including the dependence of their subnucleolar localization and rRNA-inhibitory function on the expression of Treacle^2,4,9^. Since HeV M protein appears to appropriate the DDR-Treacle pathway, we reasoned that M protein might bind at the same or overlapping site(s) in Treacle as NBS1, thereby mimicking the DDR-activated response but in the absence of DNA-damage. Such competitive binding would be consistent with the lack of additional effects of DNA-damage on rRNA biosynthesis in M-protein-expressing cells (Fig. 7d). To examine this we used IP to detect Treacle–NBS1 complexes in lysates of cells expressing mCherry, mCherry-HeV M wt or mCherry-HeV M K258A. Previous reports indicate that Treacle–NBS1 interaction is readily detected in IPs with or without DNA damage^4,8^ and, consistent with this, we detected Treacle–NBS1 complexes in co-IPs from control cells expressing mCherry alone or mCherry-HeV M K258A (Fig. 9a and Supplementary Fig. 4). However, detection of the complexes was strongly impaired in IPs from wt HeV M protein-expressing cells, indicating that HeV M protein efficiently prevents NBS1–Treacle interaction.

## Discussion

Here we identified the rRNA transcriptional regulator Treacle as a novel target for subversion of the host cell by a microbial pathogen. Treacle is a critical component in cellular mechanisms to regulate rDNA transcription following DNA damage^2,4^. The role of Treacle in this process appears to be due to interaction with NBS1, an integral player in the DDR^2,4^. Current data on the NBS1–Treacle complex are consistent with a model whereby DDR-activated NBS1 inhibits rRNA biogenesis by suppressing Treacle’s function in maintaining basal levels of rRNA (Fig. 9b left panel). Following the finding that viral proteins can bind to Treacle (Fig. 2c), we hypothesized that such a mechanism could be exploited by viruses through the formation of a complex with Treacle analogous to that formed by NBS1, thereby inducing the DDR pathway without the requirement for a DNA-damage signal (Fig. 9b right panel). Consistent with this, our data indicate that M protein interaction with Treacle displaces NBS1 from the complex, and appropriates the downstream pathway that is activated during a DDR. These data indicate that binding of specific proteins to Treacle can directly inhibit rRNA biogenesis, consistent with the idea that specific regulation of Treacle function (and/or the function of Treacle-containing complexes), rather than some alternative function of NBS1 in nucleoli/subnucleolar compartments, is responsible for rRNA suppression in the DDR. Notably, one prediction of our model is that, due to competition with M protein, NBS1 would be expected to have antiviral properties and, consistent with this, a previous genome-wide functional genomics screen of HeV infection identified NBS1 as antiviral (Z-score = 2.77)^17^. Thus, our data support both the model for DDR-mediated rRNA silencing via direct inhibition of Treacle-dependent rRNA biogenesis, and the proposed novel mechanism for viral subversion of this process.

The precise mechanisms underlying Treacle function in rRNA production are not fully resolved, but likely involve interaction with other nucleolar components critical to rDNA transcription such as RNA Pol I and the RNA Pol I transcription factor UBF1^9,36^. Intriguingly, our IP/MS analysis suggested that HeV M protein interactions within subnucleolar compartments include UBF1, indicating that HeV M protein might form complexes incorporating UBF1 and Treacle. Consistent with this, UBF1 was previously identified as an interactor of NiV M protein, although the subnucleolar localization of the interaction was not elucidated^26^. Thus, it appears that M protein interaction with the Treacle–UBF1 complex enables specific roles in the nucleolus to suppress rRNA production; indeed, targeting of the Treacle–UBF1 complex is not unexpected since Treacle’s regulation of rRNA appears to involve UBF1 interaction^9^. Importantly, however, our data using Treacle-depleted cells clearly shows that the effect of M protein on rRNA synthesis is dependent on Treacle, indicating that the targeting of Treacle specifically enables viral regulation of rRNA. This supports a role for Treacle as an organizer/regulator of rRNA synthesis in dynamic cellular responses. Notably, the interactions of Treacle, RNA Pol I, UBF1 and rDNA appear to occur within Treacle-enriched compartments (which our data and others^9,33,36^ indicate correspond to FC/DFC). This suggests that the FC/DFC co-localization facilitates specific interaction network(s) enabling roles in rRNA production and stress responses^2,4^, which our data now indicate are targeted by viruses. Delineation of the specific signaling pathways/networks that underpin the diverse functions of nucleoli and nucleolar compartments is hampered by their molecular complexity. Based on the findings of the current study with respect to NBS1–Treacle interaction, it is likely that further analysis of virus-nucleolar targeting will provide additional insights into the organization and functions of these compartments, particularly given the availability of mutated viral proteins that differ in their specific interactions with these structures.

Other than roles in rDNA transcription, Treacle also functions in pre-rRNA processing, suggesting that the nucleolar DDR pathway induced by DNA damage or by viral proteins is likely to have important effects on rRNA and ribosome function beyond suppression of rRNA synthesis. Notably, the roles of Treacle in pre-rRNA processing involve association with the Box C/D small nucleolar ribonucleoprotein (snoRNP) complex. The Box C/D snoRNP complex methylates pre-rRNA and is composed of four core proteins: FBL, NOP56, NOP58, and NHP2L1^37^. Three of these (FBL, NOP56 and NOP58) have been implicated as being important to a henipavirus infection in a genome-wide functional genomics screen^17^, and confirmed or proposed to associate with M protein. However, no direct function for these interactions has yet been demonstrated^17,26,27^ and, importantly, HeV M interaction with FBL was unaffected by K258A (Supplementary Fig. 3). Thus, the FBL interaction appears to be distinct from the role of HeV M protein in Treacle-dependent modulation of rRNA production. Taken together with the findings that other nucleolar interactors including NPM1 and NCL were unaffected by K258A mutation (Fig. 2c), that IP/MS analysis of henipavirus M protein has identified multiple additional nucleolar interactors (our data and others^26,27^), and that a functional genomics screen identified several nucleolar factors that inhibit infection^17^, this suggests that M proteins are likely to have additional nucleolar roles independent of Treacle interaction.

Notably, our data not only indicate that HeV/NiV M proteins manipulate the nucleolar DDR via M protein, but also show that infection by virus produces DNA damage. Although DNA damage/DDR modulation is well known for a number of DNA and retroviruses, roles in infection by RNA viruses, particularly mononegaviruses is less well defined^38^. The simplest interpretation of our data is that henipavirus M proteins have evolved mechanisms to modulate specific elements of the DDR enabling virus to replicate efficiently within a hostile environment. For example, M protein’s apparent modulation of the DDR in the absence of DNA damage might produce a protective environment against virus-induced DNA damage. Indeed, previous studies have indicated that NiV and other mononegaviruses, such as rabies virus, can induce reactive oxygen species (ROS), which can cause DNA damage^39,40^. Intriguingly, sequencing of bat genomes, including Pteropodid bats (the natural reservoir of HeV, NiV, lyssaviruses and many other highly pathogenic viruses) revealed that many DNA-damage checkpoint genes have been positively selected for^41^, perhaps to protect bat cells against damage by the large amounts of ROS generated during flight. It has been proposed that this might also be important to the capacity of bats to ‘tolerate’ and act as reservoirs for many pathogenic viruses^41^. Our novel identification of interactions of henipaviruses with the DDR support critical roles in the host–virus interface, that potentially differ in bats and other host species.

## Methods

### Antibodies and reagents

Rabbit anti-TCOF1/treacle was purchased from Proteintech (Cat #11003-1-AP) and used for immunofluorescence (IF; 1:100) and immunoblotting (IB; 1:2000). Mouse anti-GFP (Sigma-Aldrich, Cat #11814460001 ROCHE; 1:2000), anti-β-tubulin (Sigma, Cat # T8328; 1:2000), and anti-β-actin (Abcam, Cat# Ab3280; 1:2000) were used for IB. Mouse anti-HeV M (Generated at AAHL; 1:10)^42^ was used for IF^25^. Anti-FBL (Abcam, Cat #Ab4566), anti-NCL (CST, Cat#14574), anti-UBF1/2 (gift from Prof. Ross Hannan (Australian National University), in-house generated antibody^43^, 1:100) and anti-Phospho-Histone H2A.X (Ser139) (γH2AX; CST, Cat#2577; 1:800) were used for IF and/or immunoblotting. Secondary antibodies diluted 1:1000 were used for IF (goat anti-rabbit Alexa Fluor 568 (Cat #A-11011), goat anti-rabbit Alex Fluor 647 (Cat #A-21245)) were purchased from Thermo Fisher Scientific. Secondary antibodies diluted 1:10,000 were used for IB (goat anti-rabbit (Cat #AP307P) and goat anti-mouse (Cat #AP308P) IgG horse radish peroxidase (HRP)-conjugated-antibodies) were purchased from Merck. rRNA synthesis was inhibited by treating cells with Actinomycin D (ActD) (Thermo Fisher Scientific, Cat #11805017) for 1 h at 500 ng/mL. DNA damage was induced by treating cells with 50 μM etoposide for 3 h.

### Cell culture

HeLa (*ATCC* CCL-2) and HEK-293T (*ATCC* CRL-3216) cells were maintained in Dulbecco’s Modified Eagle Medium (DMEM, ThermoFisher Scientific, Cat# 11965092) supplemented with 10% Fetal Calf Serum (FCS) at 37 °C, 5% CO_2_.

### Transfections

Plasmids for expression in mammalian cells of HeV-M protein (Accession Number AEB21196.1), NiV-M (Accession Number AAY43914.1), and mutants thereof, fused at the N terminus to GFP or mCherry, were generated by directional cloning of M gene cDNA into the multiple cloning site of the pEGFP-C1 vector, as previously described^21^. mCherry-NCL and NBS1-GFP were kind gifts from Keiichi I. Nakayama (Kyushu University) and S. Elledge (Harvard University), respectively. siRNA targeting Treacle consisted of a pool of 3 Treacle-specific siRNAs, synthesized by Bioneer Pacific^®^ (Sequences (5′-3′): GGUCUCCAUCCAAGGUGAAA(dTdT); CAGUAGUGAGGAGUCAUCA(dTdT); GCAAGCUAAGAAAACCCGU(dTdT)).

Plasmids were transfected into HEK-293T cells and HeLa cells using Lipofectamine 2000^TM^ and Lipofectamine 3000^TM^, respectively, according to the manufacturer’s instructions (Thermo-Fisher Scientific). siRNA (100 nM final) was transfected into cells using DharmaFECT 1 Transfection Reagent^TM^ (GE Dharmacon) according to the manufacturer’s instructions.

### Confocal laser scanning microscopy and image analysis

CLSM used a Leica SP5 or Nikon C1 microscope with 60× oil immersion objective (NA 1.4), or a Leica SP8 with Hyvolution, and a heated chamber (37 °C) for live-cell analysis. Image analysis was performed using ImageJ freeware software. For IF staining, cells seeded onto glass coverslips were fixed with 4% paraformaldehyde (37 °C, 10 min), permeabilized with 0.25% Triton X-100 (room temperature (RT), 5 min), and blocked with 1% bovine serum albumin (BSA) in PBS (RT, 1 h), before primary and secondary antibody labeling (RT, 90 min each), and coverslips were mounted onto glass slides with Mowiol.

### *d*STORM imaging and analysis

For *d*STORM imaging, HeLa cells were fixed 24 h p.t. using 4% paraformaldehyde (37 °C, 10 min), and permeabilized using 0.1% Triton X-100 (RT, 10 min). Slides were then blocked with 2% BSA in PBS (RT, 30 min) before labeling using anti-Treacle (3 µg/ml, 1 h, RT) and Alexa Fluor-647-conjugated anti-rabbit secondary antibodies (5 µg/ml, 45 min, RT). Labeled cells were imaged in a switching buffer of 10% glucose, 100 mM mercaptoethylamine, 400 µg/ml glucose oxidase and 35 µg/ml bovine catalase in PBS, made to pH 8.5 using 1 M KOH^44^. Imaging was performed on home-built super-resolution set-up based on a previously described system^35^, comprising an inverted fluorescence microscope (Olympus IX81, 100 × 1.49 NA TIRF objective), using a blue laser (Toptica iBeam smart laser diode, 488 nm laser, ~50 W cm^2^) to identify GFP-positive cells in epifluorescence. A high power red laser (Oxxius Laser Box, 638 nm, 3–5 kW cm^2^) was used to induce photoswitching of Alexa Fluor-647 molecules in highly inclined laminar optical (HiLo) illumination with the sample under reducing buffer. Once single molecule ‘blinking’ events were sufficiently sparse, frames were acquired at 100 Hz (10 ms/frame) for 1–3 min on an sCMOS camera (pco. edge 4.2), as a TIF stack. Typically 10,000–20,000 frames of single molecule emissions were recorded and imported into rapi*d*STORM^45^ to localize each emission intensity profile, using a 2D Gaussian function, onto a 5 nm/pixel coordinate point map. Each final reconstructed image contained at least 2 million localizations. These images were analyzed to determine Treacle-enriched compartment area using ImageJ after first smoothing 2D super-resolution images with a Gaussian Blur (*r* = 0.5) to account for localization precision error. Images were colored based on increasing pixel density in grayscale (1–255) and subnucleolar compartments identified as regions where pixel density was greater than that in the nucleoplasm where no compartments were present by applying a pixel threshold (≤2) to remove background fluorescence. 3D *d*STORM imaging was achieved via the method of point spread function engineering by astigmatism. A cylindrical lens (*f* = 1000 mm) was placed into the imaging optical path and lateral PSF distortion as a function of axial position was calibrated by scanning (in *z*) 0.1 µm Tetraspeck spheres embedded in a water soluble gel matrix and fitting the changing lateral distortions of emission intensity with a 2D Gaussian^46^. 3D super-resolution images and movies of cells were generated using ViSP^47^ from 3D localization coordinates determined in rapi*d*STORM. Subnucleolar compartments were color-coded for relative 3D density to highlight spheroidal conformation.

### Co-immunoprecipitation (co-IP)

All co-IPs were performed using 6 cm dishes of HEK-293T cells that were transfected to express the indicated proteins and lysed at 24 h p.t. with lysis buffer (10 mM Tris/Cl pH 7.5; 150 mM NaCl; 0.5 mM EDTA; 0.5% NP-40, 1 × Protease Inhibitor Cocktail (PIC; Sigma-Aldrich Cat #11697498001)) for 30 min at 4 °C. Supernatants were collected by centrifugation at 20,000 xg for 15 min at 4 °C and 10% of the cleared lysate was collected for ‘input’ analysis; the remaining lysate was subjected to IP using 10 µL of GFP-Trap^®^ beads (Chromotek)^48^. Beads were washed 3 times with dilution buffer (10 mM Tris/Cl pH 7.5; 150 mM NaCl; 0.5 mM EDTA, 1 × PIC). Samples for IB analysis were resuspended in 2 × SDS-PAGE sample loading buffer. Samples for mass-spectrometry analysis were subjected to a final wash containing no PIC.

### Mass spectrometry and analysis

Processing of co-IP samples was performed at the Bio21 Mass Spectrometry and Proteomics facility (University of Melbourne). Proteins bound to beads from IP assays were eluted using trifluoroacetic acid (TFA) prior to readjustment of pH to ~8 with triethylammonium bicarbonate buffer (TEAB) and trypsin digestion. LC MS/MS was performed on a QExactive plus Orbitrap mass spectrometer (Thermo Scientific) with a nanoESI interface in conjunction with an Ultimate 3000 RSLC nanoHPLC (Dionex Ultimate 3000). The LC system was equipped with an Acclaim Pepmap nano-trap column (Dinoex-C18, 100 Å, 75 µm x 2 cm) and an Acclaim Pepmap RSLC analytical column (Dinoex-C18, 100 Å, 75 µm x 50 cm). Result files were searched against the SwissProt database in a target decoy fashion using MASCOT (Version 2.4.1, Matrix Science, UK). Proteins containing ≥2 significant peptides with mascot ion score greater than the identity score (*p* < 0.05) were deemed significant. Interactions identified were confirmed in ≥2 separate assays.

### VLP assay

Transfected HEK-293T cells in six-well tissue culture plates were processed 24 h p.t.; supernatant was collected and cleared by centrifugation at 1750 × *g* (10 min) in a benchtop centrifuge. Cleared supernatants were then ultracentrifuged on a 20 % (w/v) sucrose cushion at 25,000 rpm at 4 °C (16 h) with a SW41 rotor using a Beckman Coulter Optima L-90K ultracentrifuge. Pelleted VLPs were resuspended in 50 μl sodium chloride/Tris/ EDTA buffer, and then SDS-PAGE loading buffer was added. The cells were then lysed in lysis buffer (10 mM Tris/Cl pH 7.5; 150 mM NaCl; 0.5 mM EDTA; 0.5% NP-40, 1 × PIC). Lysates and VLP samples were analysed by SDS-PAGE/IB. The budding index was determined as previously^19^ for siRNA treated experiments, by measuring the intensities of the bands by densitometry using Image Lab^TM^ (Bio-Rad) software for VLP and lysate samples. The budding index was defined as the amount of M protein in VLPs divided by the amount in the cell lysate, and calculated relative to budding for siNEG transfected cells.

### SDS-PAGE and immunoblotting

Samples were separated on 8, 10, or 12% denaturing gels by SDS-PAGE before transfer to a nitrocellulose membrane using a BioRad Trans-Blot semi-dry apparatus. After blocking (5% non-fat milk in PBS with 1% Tween20 (PBST)), the membranes were incubated with primary antibodies followed by HRP-conjugated goat anti-rabbit or anti-mouse secondary antibodies, and imaged on a Gel Doc™ XR + Gel Documentation System. Uncropped scans of critical representative IBs are presented in Supplementary Fig. 5.

### 5-ethnyl uridine (EU) incorporation assays

Analysis of rRNA was performed as previously^4^ whereby determination of nascent rRNA was detected using the Click-iT^TM^ RNA Alexa Fluor 594 Imaging Kit (Thermo-Fisher, Cat# C10330). Cells were incubated for 1 h in the presence of EU before fixation in 4% paraformaldehyde at RT for 12 min, and permeabilization in 0.25% Triton X-100 for 5 min at RT. Samples were then processed according to the manufacturer’s recommendations to label incorporated EU with Alexa Fluor 594. Cells were imaged by CLSM to detect labeling of nascent rRNA by measuring the fluorescence intensity of Alexa Fluor 594 within nucleoli. Quantitative analysis to determine EU fluorescence (arbitrary units, a.u.) was performed using ImageJ software, with nucleoli identified using differential interference contrast (DIC) microscopy and IF labeling of nucleoli by anti-FBL or anti-Treacle/TCOF1.

### Virus infections

Wild type HeV (Hendra virus/horse/1994/Hendra) was used for all virus work. All work with infectious virus was conducted at the CSIRO Australian Animal Health Laboratory (AAHL) in Biosafety Level (BSL)-4 laboratories. For analysis by IF microscopy, HeLa cells were seeded onto coverslips and mock- or HeV-infected (MOI 5) prior to fixation using 4% paraformaldehyde and permeabilization with 0.25% Triton X-100. IF labeling was performed using antibody to HeV M alone or together with antibody to Treacle, followed by Alexa Fluor^®^ 488 (for HeV M) and Alex Fluor^®^ 568 (for Treacle) secondary antibody. For EU analysis, HeLa cells were seeded into 8-well chambers and mock or HeV-infected (MOI 5). EU was added to media 1 h prior to fixation at 24 h p.i. with 4% paraformaldehyde, and analysis as described above. In all cases, cells were decontaminated for 2 h using 4% paraformaldehyde before removal from BSL-4 laboratories.

### Tissue culture infective dose (TCID_50_) analysis

HeLa cells were transfected without siRNA, or with scr siRNA, Treacle siRNA or PLK1 siRNA (3 days) prior to infection with HeV at MOI 0.5. siRNA to PLK1, which induces apoptosis and cell death, was used as a transfection and indirect positive control^17^. Viral TCID_50_ was determined as described previously^49^, in which samples were titrated in triplicate in 96-well plates and co-cultured with Vero cells for 3 days. The infectious titer was calculated by the method of Reed and Muench^50^.

### Data availability

The authors declare that the data supporting the findings of this study are available within the article and its Supplementary Information files, or are available from the corresponding author upon request.

## Electronic supplementary material

Supplementary Data 1

Supplementary Information

Description of Additional Supplementary Files

Supplementary Movie 1

Supplementary Movie 2
