# Supplementary material for: Viral regulation of host cell biology by hijacking of the nucleolar DNA-damage response
Source: Nat Commun. 2018 Aug 3;9:3057. doi: 10.1038/s41467-018-05354-7 (PMC6076271; doi:10.1038/s41467-018-05354-7)
Supplement: Supplementary file 2 — Supplementary Information [file 41467_2018_5354_MOESM2_ESM.pdf]

Viral hijacking of the nucleolar DNA-damage response is a novel mechanism to regulate host biology

Rawlinson et al.

Supplementary Information

Supplementary Figure 1

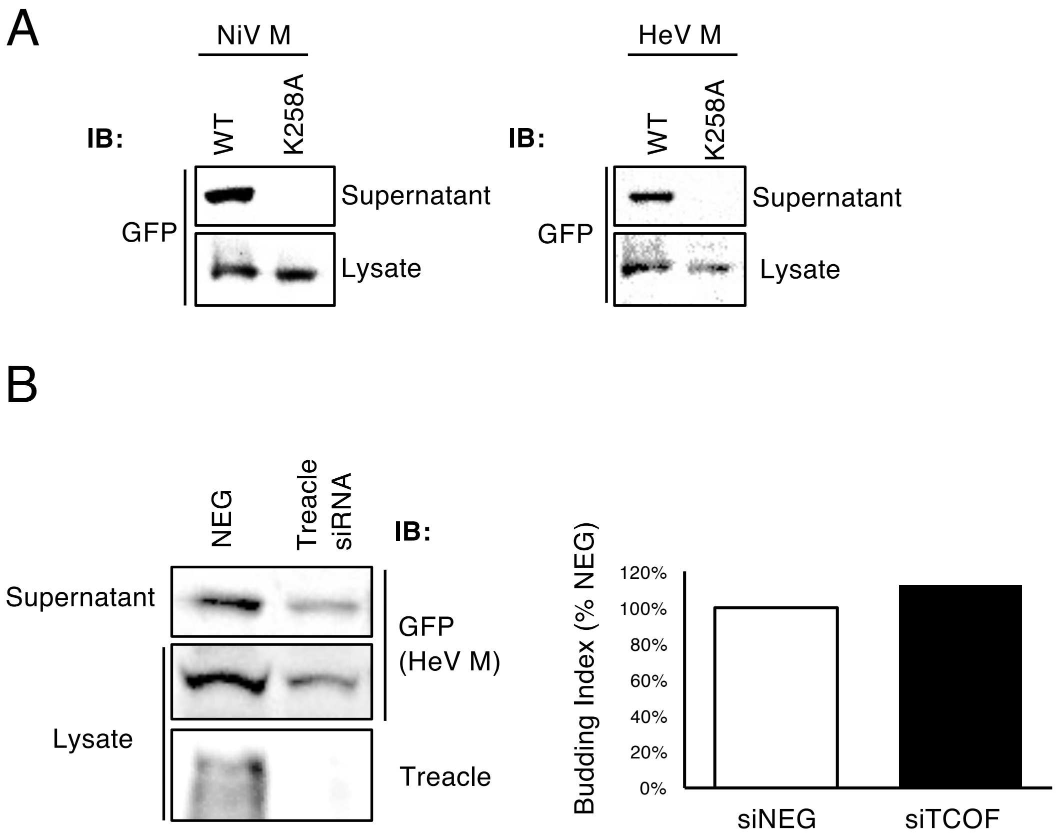

Supplementary Figure 1. **K258A mutation but not Treacle depletion reduces VLP formation by HeV M protein**. (A) HEK-293T cells were transfected to express GFP-tagged NiV M protein (left panel) or HeV M protein (right panel). 24 h p.t. VLPs (supernatant) and cell lysates were harvested and analyzed by IB using anti-GFP antibody. (B) HEK-293T cells were transfected with Treacle siRNA or negative control (NEG) two days prior to transfection to express GFP-HeV M protein. VLPs and lysates were harvested 24 h later and analyzed by IB to determine the budding index (right panel; see Materials and Methods); data are representative of two independent assays.

Supplementary Figure 2

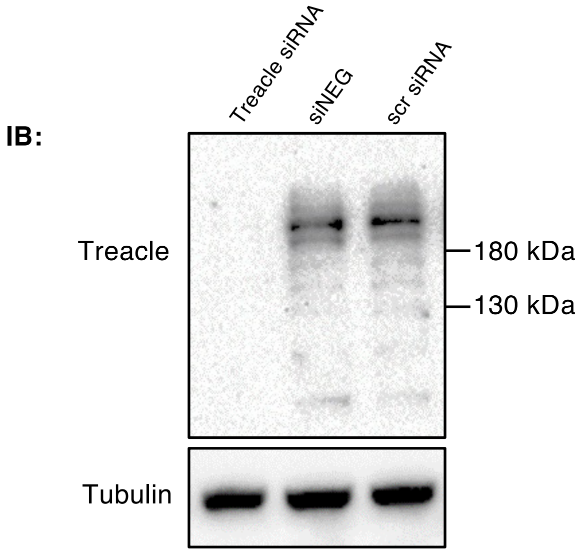

Supplementary Figure 2. siRNA knockdown reduces expression of Treacle protein in HeLa cells. HeLa cells were transfected without siRNA (NEG) or with scr siRNA or Treacle siRNA before lysis 3 days p.t. and analysis by IB using antibodies to Treacle and tubulin. A predominant ~220 kDa band corresponding to Treacle was detected, as well as several isoforms in lysates of control cells, but not of Treacle siRNA-transfected cells.

Supplementary Figure 3

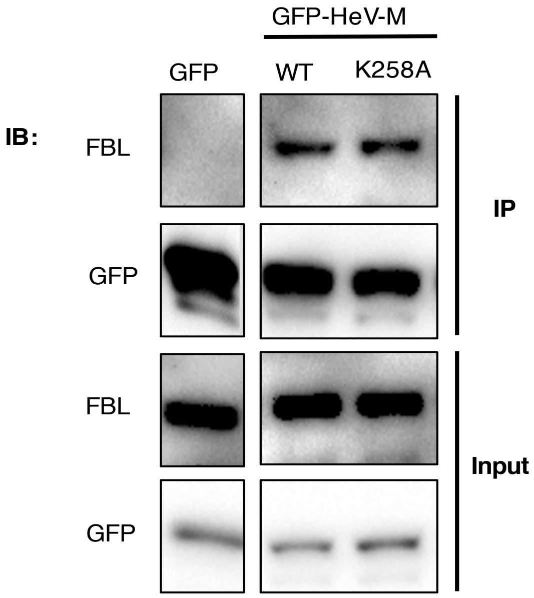

Supplementary Figure 3. GFP-HeV M wt and K258A proteins interact with fibrillarin. HEK-293T cells transfected to express the indicated GFP-fused proteins were subjected to IP for GFP before analysis by IB using the indicated antibodies.

Supplementary Figure 4

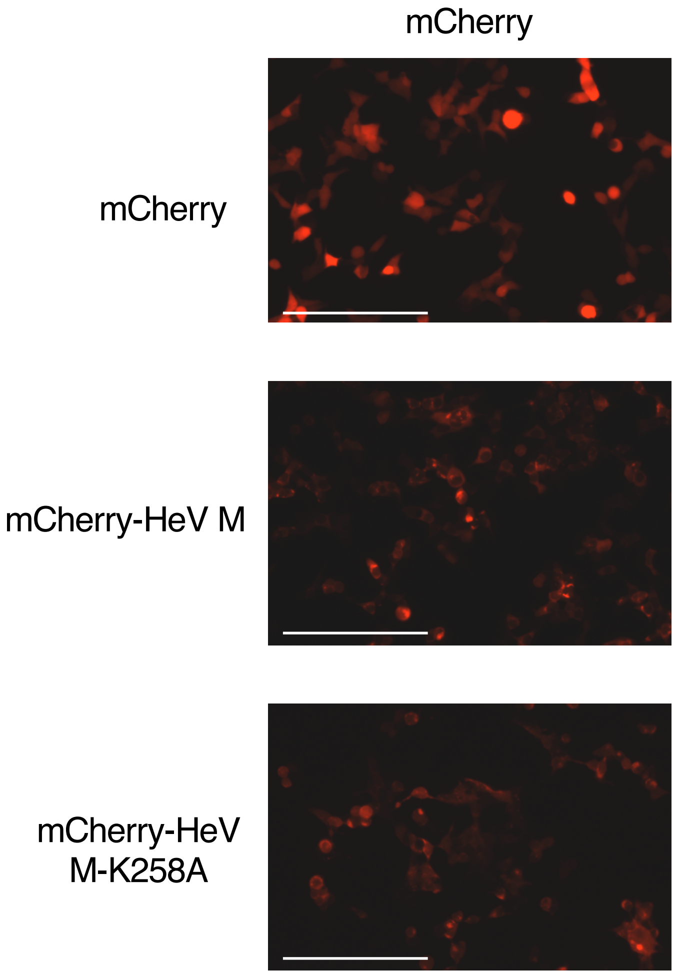

Supplementary Figure 4. Microscopy analysis of mCherry protein expression. HEK-293T cells used to generate samples shown in Figure 9A were imaged using an EVOS FL Cell Imaging System (Thermo Fisher Scientific) immediately prior to cell lysis. Scale bars correspond to 200 μm.

Supplementary Figure 5

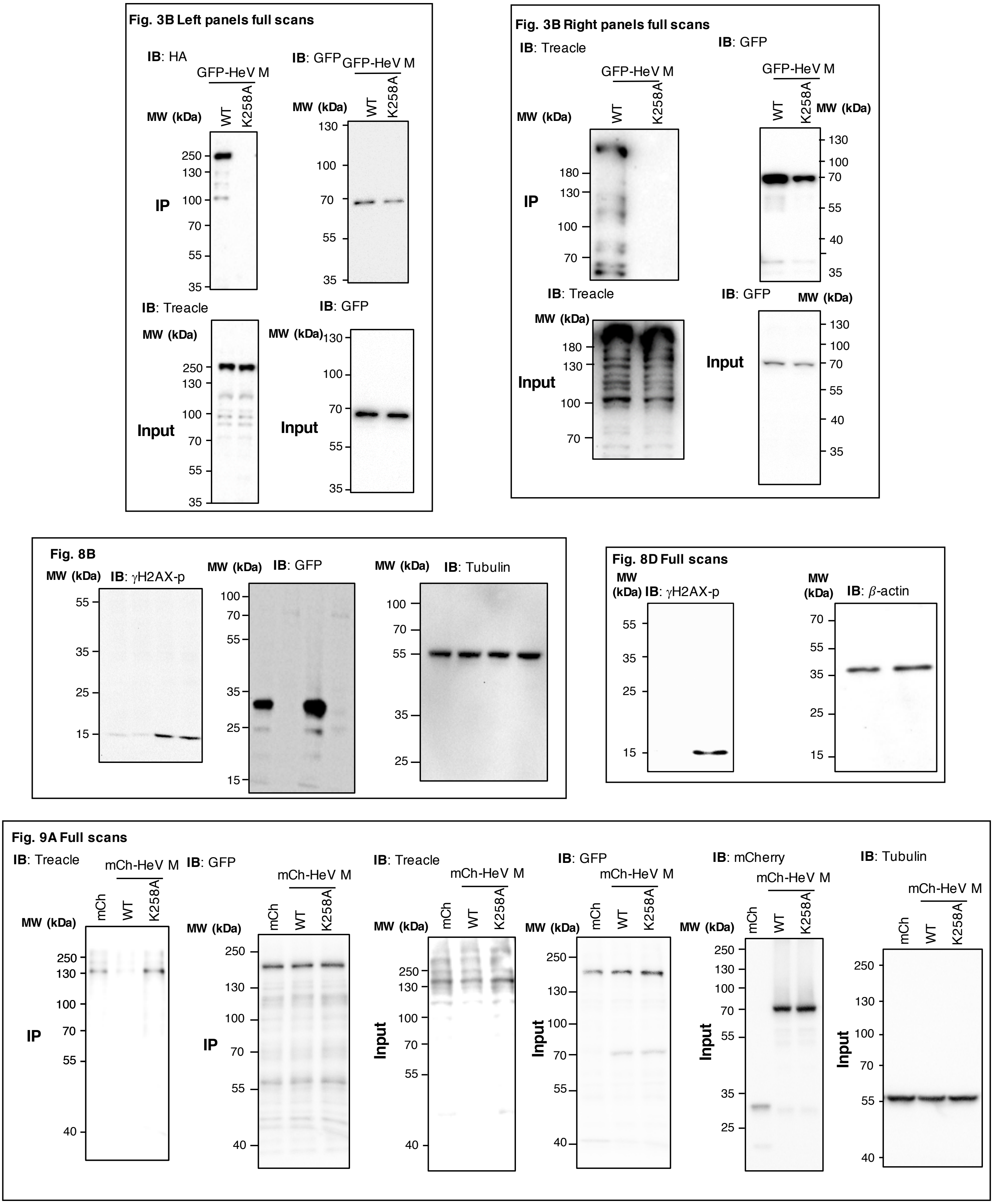

Supplementary Figure 5 Full scans of immunoblots shown in Results.
