# Supplementary material for: Viral regulation of host cell biology by hijacking of the nucleolar DNA-damage response
Source: Nat Commun. 2018 Aug 3;9:3057. doi: 10.1038/s41467-018-05354-7 (PMC6076271; doi:10.1038/s41467-018-05354-7)
Supplement: Supplementary file 3 — Description of Additional Supplementary Files [file 41467_2018_5354_MOESM3_ESM.pdf]

Description of Additional Supplementary Files

File Name: Supplementary Movie 1
Description: Video of a 3D image of a living HeLa cell expressing GFP-HeV M and RFP-FBL (nucleolar marker). Images were acquired using a Leica SP8 microscope with Hyvolution (pinhole at 0.5 Airy units), using 4 x line averages with high sensitivity HyD detectors, and then deconvolved using Huygens (SVI, Netherlands).

File Name: Supplementary Movie 2
Description: Video generated using the 3D *d*STORM image shown in Fig. 5A.

File Name: Supplementary Data 1
Description: Proteins identified using mass spectrometry.
HeV M wt or HeV K258A M were immunoprecipitated from HEK-293T cells before analysis of interactome by MS as described in materials and methods.
